# Uterine Lipoleiomyoma Presenting With Pelvic Pain in a Post-Menopausal Woman

**DOI:** 10.7759/cureus.14929

**Published:** 2021-05-10

**Authors:** Fidel S Rampersad, Shiva Verma, Jason Diljohn, Vashisht Persad, Prakashbhan Persad

**Affiliations:** 1 Radiology Unit, Department of Clinical Medical Sciences, The University of the West Indies, St. Augustine Campus, Port of Spain, TTO; 2 Department of Obstetrics and Gynecology, The University of the West Indies, St. Augustine Campus, Port of Spain, TTO; 3 Department of Radiology, The University of the West Indies, St. Augustine Campus, Port of Spain, TTO; 4 Department of Obstetrics and Gynecology, Maimonides Medical Center, Brooklyn, USA; 5 Department of Obstetrics and Gynecology, Sanjivani Women’s Hospital, St. Augustine, TTO

**Keywords:** uterine lipoleiomyoma, uterine leiomyoma, fibroids, dermoid, mature ovarian teratoma

## Abstract

Uterine leiomyomas (fibroids) are the most common tumor of the reproductive system in women between menarche and menopause. Uterine lipoleiomyomas are a rare variant of leiomyoma, consisting of smooth muscle cells admixed with adipocytes.

Herein is the case of a 70-year-old female who presented with acute pelvic pain and a palpable pelvic mass. A computed tomography scan of her abdomen and pelvis demonstrated a large, circumscribed, fat and soft tissue density, uterine mass suggestive of a lipoleiomyoma. Histopathology examination of the resected specimen after total abdominal hysterectomy confirmed a mature lipoleiomyoma.

## Introduction

Uterine lipoleiomyoma (LLM) is a rare variant of leiomyoma occurring with a frequency between 0.03% and 0.2% and predominantly found in an intramural location [1-3]. It is a benign, lipocyte rich uterine tumor, most prevalent in obese perimenopausal or postmenopausal women [4]. Histologically, LLMs are composed of variable amounts of smooth muscle, adipocytes and fibrous tissue. The large majority of patients are asymptomatic. In younger patients however, a common presentation is a painful pelvic mass with micturition difficulties and menstrual disturbances [3]. Treatment of LLM ranges from medical management to surgery.

## Case presentation

**Figure 1:**
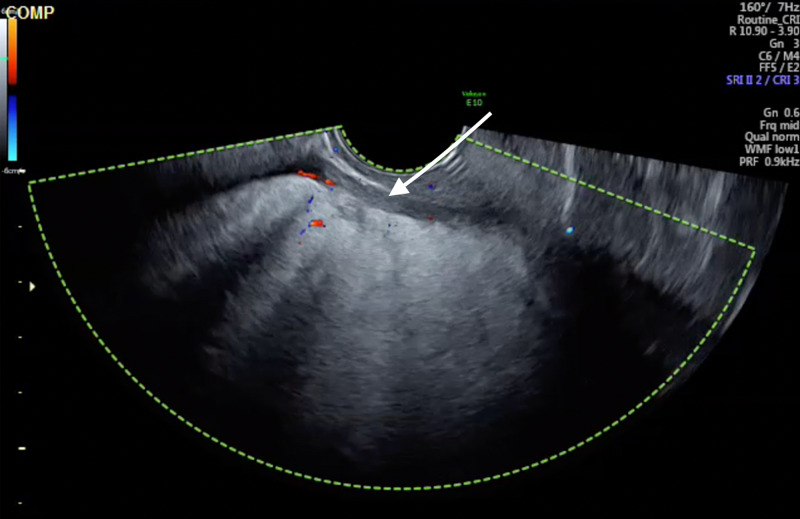
Transvaginal pelvic ultrasound image with colour Doppler revealing a markedly echogenic midline pelvic mass with minimal internal vascularity (white arrow).

A 70-year-old, gravida 4 para 4, previously well postmenopausal woman, presented to her gynaecologist with sudden onset pelvic pain. There was no history of acute vaginal bleeding, dysuria or hematuria. Her last menstrual period was at age 50. Past surgical history included abdominoplasty. On abdominopelvic examination, there was a 12/40 midline pelvic mass. Pelvic ultrasonography revealed a 8-cm markedly hyperechoic pelvic mass, likely of uterine origin, and no ascites (Figure 1).

**Figure 2:**
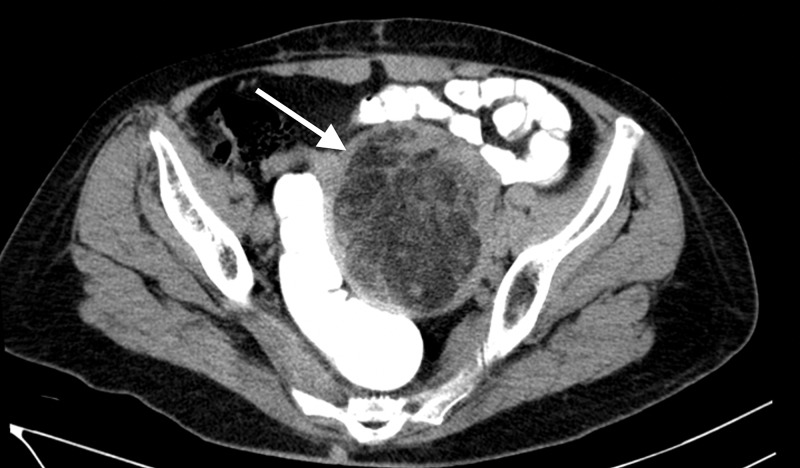
Axial CT image through the pelvis, with rectal contrast, revealing a circumscribed pelvic mass containing fat and soft tissue density arising from the uterus (white arrow).

**Figure 3:**
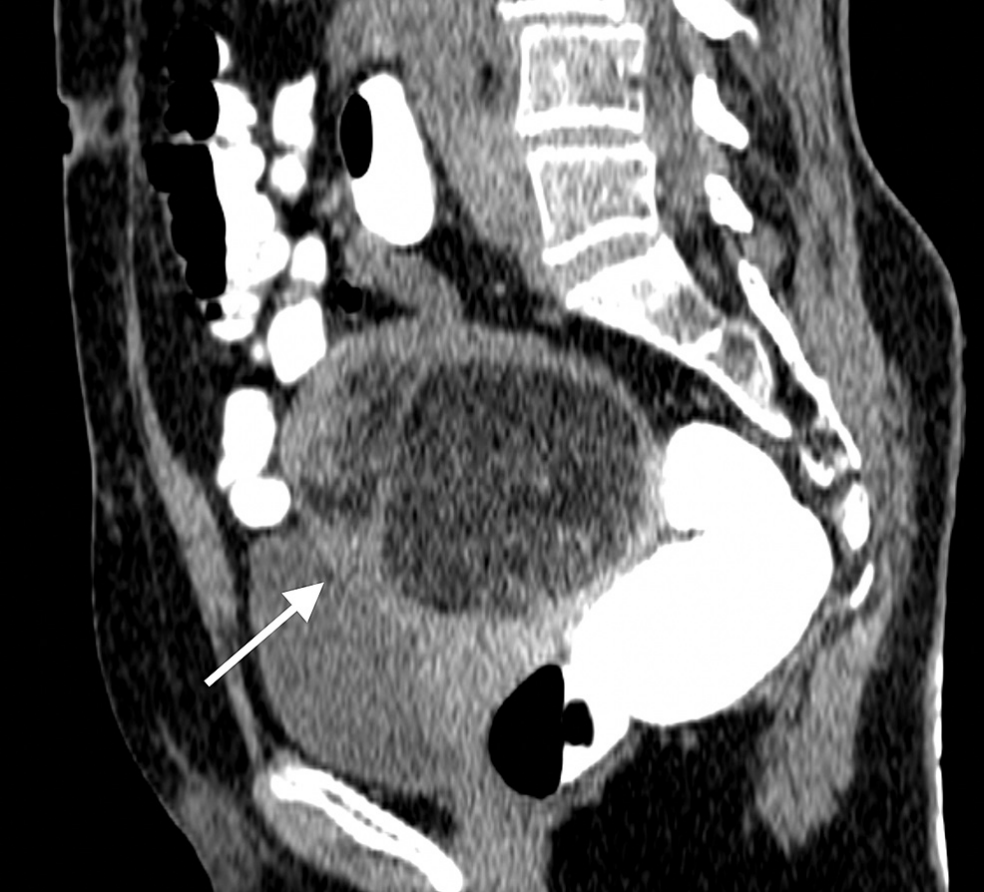
Sagittal CT reformat image with rectal contrast, showing the fat and soft tissue density uterine mass (white arrow) located anterior to the rectum and indenting the posterior wall of the urinary bladder.

**Figure 4:**
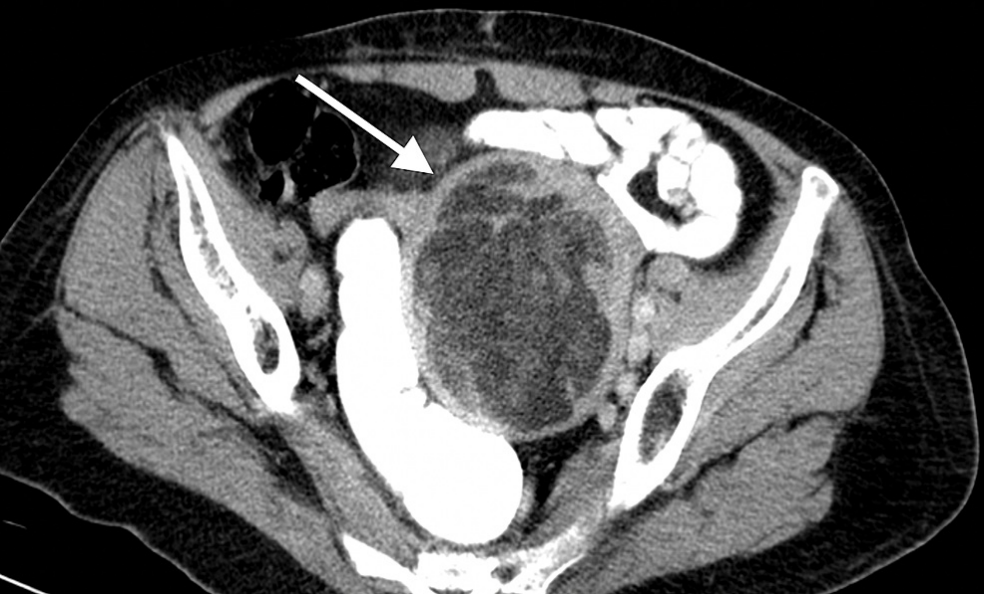
Post intravenous contrast administration, the uterine mass (white arrow) demonstrated normal peripheral myometrial enhancement with no significant enhancement of its central portion.

A computed tomography (CT) scan of her abdomen and pelvis was subsequently done and demonstrated a large, circumscribed, fat and soft tissue density, uterine mass measuring 8.9 x 7.6 x 8.5 cm, abutting the left pelvic side wall, subjacent small bowel loops, left lateral border of the recto-sigmoid colon and posterior wall of the urinary bladder (Figures 2-4). The uterine cervix and vaginal vault were normal. There was no ascites, omental cake, peritoneal deposits or abdominopelvic lymphadenopathy. Tumor markers including Cancer Antigen 125 (CA-125), beta human chorionic gonadotropin (beta-hCG), alpha fetoprotein (AFP) and carcinoembryonic antigen (CEA) were normal.

**Figure 5:**
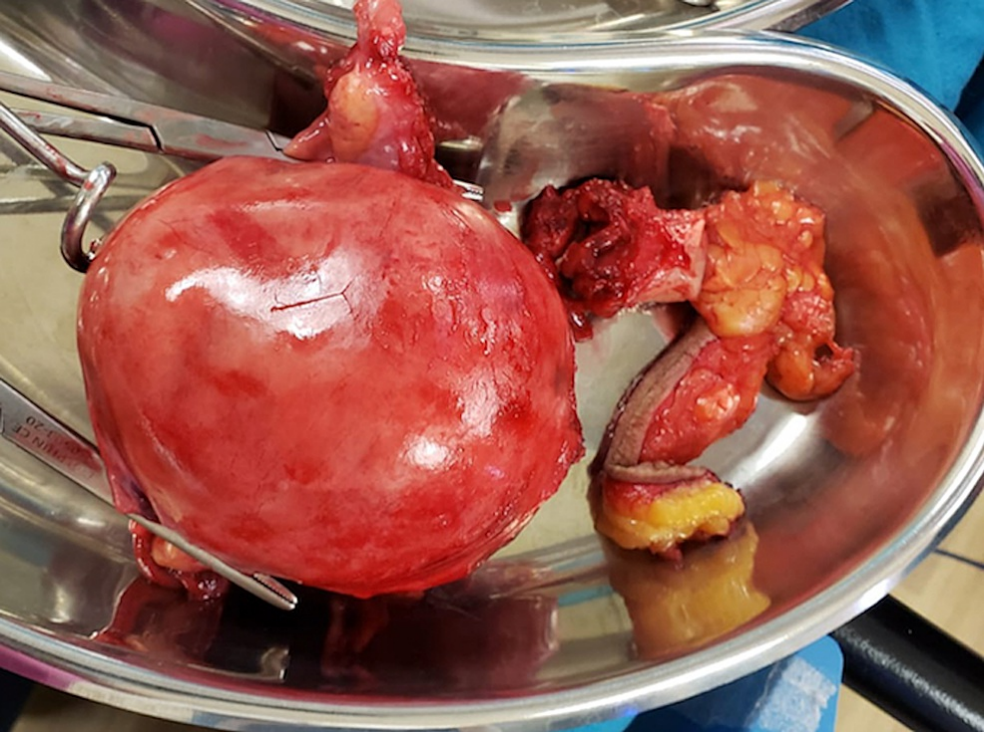
Specimen photograph showing excised uterine mass.

**Figure 6:**
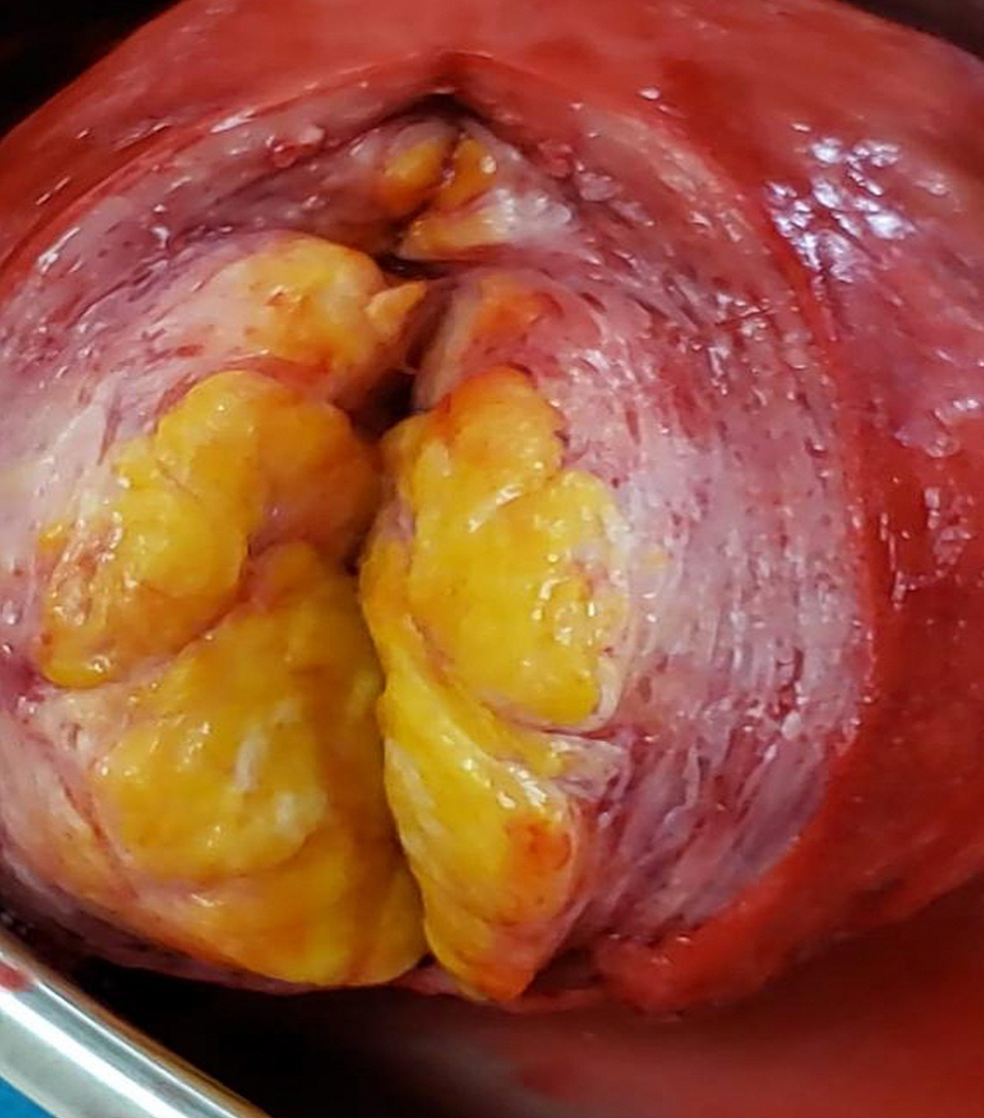
Fat (yellow) and soft tissue (pink) components of uterine mass.

**Figure 7:**
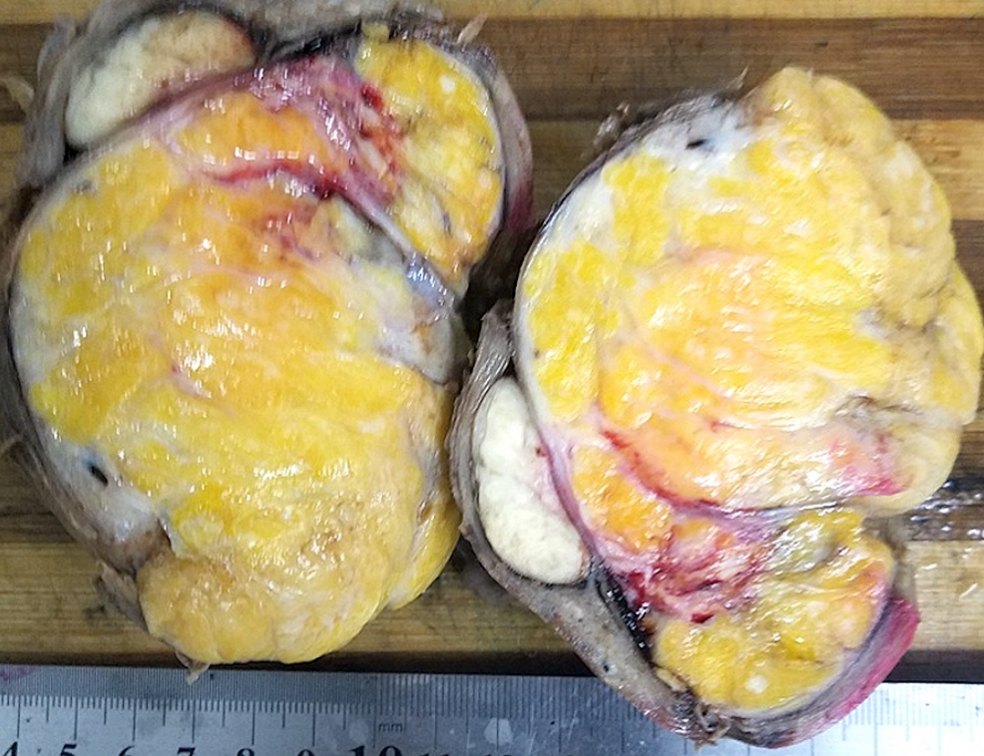
Photograph of cut section of the uterine mass showing a nodular yellow to grey white fatty consistency.

**Figure 8:**
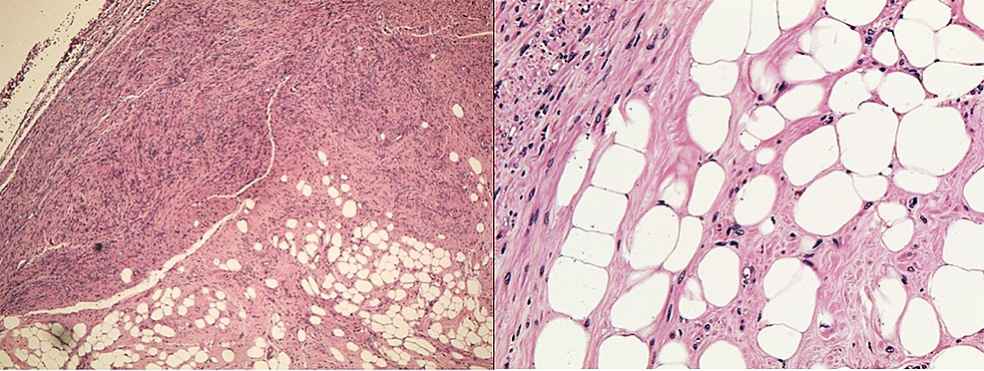
Microscopic images demonstrating a mixture of mature adipocytes and fibrous tissue within the uterine mass.

Gross pathology after total abdominal hysterectomy (TAH) and omental biopsy showed a uterine mass measuring 11.0 x 9.0 x 8.0 cm, with normal adnexa and fallopian tubes (Figure 5). Cut sections showed nodular yellow to grey white masses compressing the endometrial cavity (Figure 6). Upon further dissection, the resected tumor consisted of a mixture of smooth muscle and lipocytes (Figure 7). Both fallopian tubes and ovaries were normal. The cervix showed nabothian cysts and chronic cervicitis. Omental biopsy revealed normal tissue. Histology of the uterine mass (Figure 8) showed a mixture of fibrous tissue and mature adipocytes, consistent with a lipoleiomyoma. There was no evidence of malignancy. The patient is currently well and has complete resolution of her symptoms.

## Discussion

LLM is a rare variant of leiomyoma, with two main theories of origin suggested viz a multipotential Mullerian cell origin and adipose metamorphosis of uterine smooth muscle cells [5]. LLM may occur as a solitary mass or multiple lesions, with size variations ranging from a few millimeters to about 32 cm [6]. In addition to the uterus, LLMs have been reported in other parts of the female reproductive system including the cervix, ovary, and broad ligament [7,8]. LLMs have been linked to lipid estrogen deficiency, adenomyosis, typical and atypical endometrial hyperplasia, endometriosis, polyps, and gynaecologic malignancies [8]. Fukunaga described lipomatous metaplasia of leiomyomas as the cause for LLM, a hypothesis he based on immunohistochemical findings [9]. Despite these hypotheses, however, the exact pathogenesis of LLM remains unclear.

LLM is generally asymptomatic, but can present with a range of clinical symptoms such as acute pain, abnormal vaginal bleeding, infertility, and pressure on the urinary bladder and/or rectum. Radiological imaging is crucial in detecting LLM. Ultrasonography demonstrates a markedly hyperechoic pelvic mass while CT scans show a solid mass arising from the uterus with areas of fat and soft tissue density. MRI is a useful technique, showing areas of high signal intensity on T1-weighted images which lose signal on fat suppressed sequences [6].

LLMs only require treatment if symptomatic. It is crucial however to differentiate LLM from other diagnoses which may require a different management approach and possible surgical intervention. Among the differential diagnoses, LLM is most commonly misdiagnosed preoperatively as a mature ovarian teratoma [10]. Other differential diagnoses include benign lipomas and liposarcoma [11]. When symptomatic, LLM is usually managed surgically by hysterectomy. Other treatment options include uterine artery embolization or myomectomy, depending on the extent and severity of the patients’ symptoms, age, surgical history, the presence of other leiomyomas, desire for fertility, and the location of the mass.

The pre-operative differentiation of benign variants, such as leiomyomas and LLM, from malignant tumours such as leiomyosarcoma (LMS) is challenging. While malignancy in fibroids tends to be rare, it is of great clinical importance as benign tumours may be treated conservatively or with minimally invasive techniques, which are not appropriate in the setting of malignancy. This is further complicated by the existence of intermediate variants (e.g., smooth muscle tumor of uncertain malignant potential [STUMP]) or more aggressive histologic subtypes of LMS. Some studies have looked at different features on MRI (such as varying signal areas on T2-weighted imaging [T2WI] and diffusion-weighted imaging [DWI]) and other imaging modalities [12]. Another avenue is the use of biomarkers such as serum lactic acid dehydrogenase (LDH), which is hypothesized to increase with a high rate of internal degeneration of the tumour, as is commonly seen in LMS [13]. This has shown some promise and has been recently incorporated into pre-operative scoring systems, such as the PREoperative Sarcoma Score (PRESS) system [14]. There is however currently no consensus on the usefulness of these imaging criteria or biomarkers, and they currently remain experimental.

## Conclusions

Uterine LLM is an uncommon benign uterine tumor that occurs primarily in postmenopausal females and consists of smooth muscle cells admixed with a significant amount of lipocytes. Imaging plays a pivotal role in the preoperative characterization and localization of the mass, demonstrating its adipocyte-rich consistency. Symptomatic patients with uterine LLM are generally managed with surgery. Histopathological analysis of the resected specimen confirms the presence of fibrotic tissue and mature adipocytes, and also excludes other important differentials, including liposarcoma.
